# Association between Toll-like receptor gene polymorphisms and risk of Helicobacter pylori infection: A protocol for systematic review and meta-analysis

**DOI:** 10.1097/MD.0000000000025729

**Published:** 2021-05-07

**Authors:** Xiaocong Ma, Liying Lu, Yan Tang, Weisheng Luo, Jianxiang Li, Meiwen Tang

**Affiliations:** a Graduate School, Guangxi University of Chinese Medicine; b Department of Gastroenterology, Ruikang Hospital Affiliated to Guangxi University of Chinese Medicine; c Department of Cardiology, Ruikang Hospital Affiliated to Guangxi University of Chinese Medicine, Nanning, Guangxi, China.

**Keywords:** case-control study, gene polymorphisms, *Helicobacter pylori*, meta-analysis, toll-like receptor

## Abstract

**Background:**

There were many case-control studies performed the association between TLRs gene polymorphisms and the correlation of *Helicobactor pylori* infection, these results were inconformity. Therefore, a comprehensive meta-analysis was performed to evaluate the TLRs gene polymorphism and susceptibility to *H. pylori* infection.

**Methods:**

Eligible studies were searched from PubMed, EMBASE, Web of science, Cochrane library, CNKI, CBM, Wan Fang Database and VIP Database, all the databases were searched from inception to December 2020. OR with the corresponding 95% CI were presented as associations between certain TLR gene polymorphism and the risk of *H. pylori* infection, all the included data will be analyzed with the software of Review Manager 5.2 and STATA 14.2.

**Results:**

This study will provide a high-quality evidence to find the TLR gene polymorphisms with *H. pylori* infection susceptibility.

**Conclusion:**

This study will explore which TLR genotype increase the risk of *H. pylori* infection.

## 1 Introduction

*Helicobacter pylori* (*H. pylori*) is a major gastric pathogen that infects approximately half of the world's population.^[1]^*H. pylori* infection is associated with the development of numerous gastric pathologies such as atrophic gastritis, intestinal metaplasia and gastric cancer.^[2,3]^ The susceptibility of an individual to these clinical outcomes is remain largely unknown, however, the course of infection likely depends on the *H. pylori* virulence factors, environmental factors, the genetic susceptibility of the host and the reactivity of the host immune system.^[4,5]^*H. pylori* infection is followed by DNA damage, the repair processes and increased rate of mutations, which accelerate the development of *H. pylori*-related gastric cancer.^[6–11]^

Toll-like receptors (TLRs) are cell transmembrane and pathogen-associated molecular pattern (PAMP) receptors that play an important role in the recognition of *H. pylori*, the activation of the first line of immunity against *H. pylori*.^[12]^ So far, 10 members of TLR family have been found in human body, each member has the ability to recognize specific microbial pathogens and the initiation of innate immunity.^[13,14]^ Recent years, many case-control studies performed the association between TLR gene polymorphisms and the susceptibility of *H. pylori* infection. However, limited statistical power to detect small-effect single nucleotide polymorphisms (SNPs) and the results were in conformity. To further evaluate the relationship between TLR gene polymorphisms and the risk of *H. pylori* infection, a meta-analysis was conducted in our present study.

## 2 Objective

The objective of this study was to comprehensively evaluate the TLR gene polymorphisms associated with *H. pylori* infection susceptibility.

## 3 Methods

The authors conducted this systematic review and meta-analysis protocol according to the Preferred Reporting Items for Systematic Review and Meta-analysis Protocol Guidelines (PRISMA-P).^[15]^ The protocol was registered on International Platform of Registered Systematic Review and Meta-analysis Protocols (INPLASY202130009).

### 3.1 Eligibility criteria

#### 3.1.1 Types of studies

Case-control study design in human, peer reviewed publication. provide the available genotype/allele frequency between cases and controls, the *P* value of Hardy–Weinberg equilibrium (HWE) test in the control group was greater than.05. For duplicate publications, family-based association studies, case reports or case series studies, no available genotype/allele frequency, abstracts, reviews, comments, letters and conference presentations were excluded.

#### 3.1.2 Participants

Participants affected by *H. pylori* infection and were taken serum samples will be included in the meta-analysis. Healthy population served as controls. No restrictions were placed on age, gender, country.

#### 3.1.3 Outcome

*H. pylori* infection risk comparisons.

### 3.2 Information sources

#### 3.2.1 Electronic searches

We searched the following seven databases: PubMed, EMBASE, Web of science, Cochrane library, China National Knowledge Infrastructure (CNKI), China Biology Medicine (CBM), Wan Fang Database for Chinese Technical Periodicals and VIP Database, all the Database were searched from inception to December 2020.

#### 3.2.2 Search strategy

The search strategy will use a combination of MESH terms and free terms. Details of the PubMed search strategy appear in Supplementary Materials 1.

### 3.3 Data extraction and quality assessment

#### 3.3.1 Data extraction

**Figure 1:**
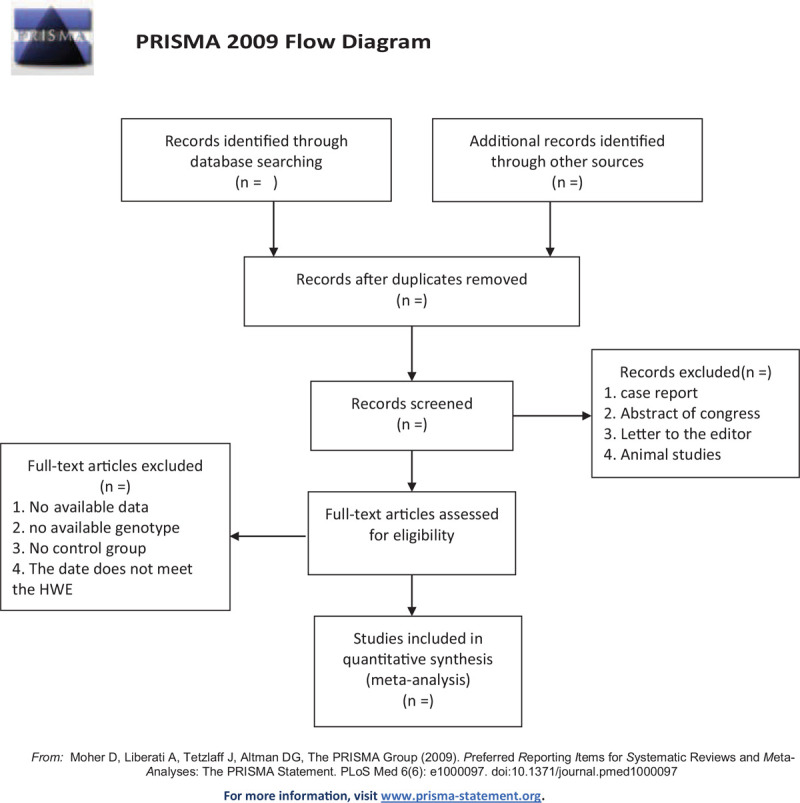
Screening process.

The extraction work were independently by two investigators (LL and YT), for disagree evaluation, an agreement was reached following consultation and discussion, the data information of all studies included: name of the first author, year of publication, country, ethnicity, sample size/age of the cases and controls, and genotype/allele frequency between cases and controls, HWE were based on genotype frequency of certain TLR gene polymorphisms in control groups, no minimum number of samples defined for inclusion in our meta-analysis. The screening process was shown as Figure 1.

#### 3.3.2 Quality assessment

The quality of included studies was assessing by the Newcastle–Ottawa scale (NOS).^[16]^ We assessed it from the selection, the comparability, and the exposure in both cases and controls, the NOS score vary from zero to nine, in which (score 0–5) was low quality study and (score 6–9) was high quality study.

### 3.4 Statistical analysis

All statistical analyses were performed by Review Manager 5.2 version software (The Cochrane Collaboration, Software Update, Oxford, United Kingdom). OR with the corresponding 95% CI were presented as associations between certain TLR genetic polymorphism and the risk of *H. pylori* infection in the allele, codominant, dominant and recessive models, the significance was determined by Z-test, with a *P* value < .05 as significant level.

### 3.5 Heterogeneity assessment

The heterogeneity assumption was checked by Q-test and I^2^ statistics, when *P* value of Q test was greater than 0.1 or I^2^ was less than 50%, fixed effects model was used for meta-analysis, otherwise, random effects model was carried out. If there were high heterogeneity, we will be found the potential sources of heterogeneity by using the methods of sensitivity analysis and subgroup analysis.

### 3.6 Subgroup analysis

We will conduct a subgroup analysis of the TLR gene polymorphisms most associated with *H. pylori* infection by ethnicity or country of participants.

### 3.7 Sensitivity analysis

Sensitivity analysis were performed by deletion of each single study in the meta-analysis to reflect the influence of the individual data-set on the pooled OR.

### 3.8 Publication bias assessment

The publication bias will be evaluated by using the funnel plots combine with statistical test(Egger and Begg test), and the Egger and Begg test will be performed by STATA software with version 14.2 (Stata Corp, College Station, USA).

## 4 Discussion

*H. pylori* infection may be related to the age, gender, race, genetic factors, geographical location and socio-economic status of the host.^[17,18]^ TLR is the main component of innate immune system and its expressed in different cell types recognize *H. pylori* through different signal pathways to activate the immune system in vivo.^[19]^ In recent years, researchers have paid more attention to the influence of host genetic factors on *H. pylori* susceptibility.^[20,21]^ Recent years, many case-control studies performed the association between TLR gene polymorphisms and the susceptibility of *H. pylori* infection, but there is no consensus in the literature. Therefore, it is necessary to conduct a systematic review to assess the TLR gene polymorphisms and the risk of *H. pylori* infection.

## Author contributions

**Conceptualization:** Weisheng Luo.

**Data curation:** Liying Lu.

**Methodology:** Yan Tang.

**Project administration:** Meiwen Tang.

**Resources:** Jianxiang Li.

**Writing – original draft:** Xiaocong Ma.

## Supplementary Material

Supplemental Digital Content
