# Supplementary material for: Association between Toll-like receptor gene polymorphisms and risk of Helicobacter pylori infection: A protocol for systematic review and meta-analysis
Source: Medicine (Baltimore). 2021 May 7;100(18):e25729. doi: 10.1097/MD.0000000000025729 (PMC8104181; doi:10.1097/MD.0000000000025729)
Supplement: Supplemental Digital Content [file medi-100-e25729-s001.doc]

(#1:“Helicobacter pylori”[Mesh] OR “H. pylori”[Title/Abstract], #2:(“Toll-Like Receptors” [Mesh] OR “TLR” [Title/Abstract]), #3(“Polymorphism, Genetic” [Mesh] OR “variant” [Title/Abstract] OR “genotype” [Title/Abstract])

#1 and #2 and #3
